# ForNet: classification of critical forest acoustic events using discriminative CNN representations and ensemble learning

**DOI:** 10.1038/s41598-026-55380-5

**Published:** 2026-07-09

**Authors:** Deepak Krishnamoorthy, Vemulapalli Shanmukha Sai, R. Vishal, Yannick Benezeth

**Affiliations:** 1 Department of Computer Science and Engineering, Amrita School of Computing, Amrita Vishwa Vidyapeetham, Chennai, 601103 India; 2 Department of Mechanical Engineering, Amrita School of Engineering, Amrita Vishwa Vidyapeetham, Chennai, 601103 India; 3 ImViA UR7535, Université Bourgogne, Europe, Dijon, France

**Keywords:** Computational biology and bioinformatics, Engineering, Mathematics and computing

## Abstract

Various studies have been conducted in the field of sound event classification, with a specific emphasis on urban environments. However, there has been limited investigation in the realm of forest-oriented sound event classification. We introduce FSM5, a task-specific forest acoustic dataset curated from multiple publicly available sources to support forest sound event classification research, and propose a two-stage framework, ForNet, for effectively classifying critical sound events in forest. Initially, a Convolutional Neural Network (CNN) is used to extract discriminative audio embeddings. In the subsequent phase, ensemble classifiers such as XGBoost and Random Forest are employed for classification. The performance of MFCC, Log-Mel, and Mel spectrogram features is systematically evaluated, and the results indicate that MFCC and Log-Mel features significantly enhance classification performance. The findings indicate that combining handcrafted acoustic representations with CNN-derived embeddings yields improved performance compared to end-to-end CNN classification. The efficacy of deep CNN’s feature representation and the discriminative capability of shallow classifiers are evaluated. To showcase the reliability of ForNet, we have also conducted tests on the benchmark: Urbansound8k dataset. ForNet achieves an accuracy of 94% under 10-fold cross-validation on UrbanSound8K, outperforming several state-of-the-art methods, and attains an accuracy of 91.4% using 10-fold cross-validation on the FSM5 dataset.

## Introduction

Audio categorization is an essential outcome of signal processing and has a substantial influence in various domains, such as entertainment and security. This technology’s significance lies in its ability to categorize audio data and its potential to enhance and optimize other technologies, such as video surveillance systems. Especially in situations when there are frequent camera blackouts or areas without surveillance coverage, audio categorization becomes an essential tool for inference, allowing for a cost and memory-effective alternative method of monitoring and analysis. Audio classification involves the identification and categorization of different auditory stimuli, which can range from murmurs to loud and sudden noises. Its main objective is to compensate for visual disturbances and provide important information about the surrounding environment, events, and potential hazards. Using audio classification under video blackout conditions enhances surveillance capabilities and highlights its wider usefulness in ensuring comprehensive situational awareness and security. This emerging discipline shows great potential, especially in specific areas like, Urban/household safety^1,2^, road safety^3^ and forest monitoring^4^, where accurately identifying and categorizing sounds is crucial for protecting natural ecosystems.

Environmental sound recognition (ESR) plays a vital role in monitoring and detecting acoustic events in the wild. Recently, numerous approaches have been proposed with various techniques coupled with edge devices to protect the reserved forests from natural and man-made disasters. Among the threats to forests are wildfire, illegal logging, and the hunting of endangered species^4–8^. The way researchers classify anomalous sounds has changed over time. In the recent past, machine learning algorithms like K-nearest neighbors (KNN)^9^, Gaussian mixture modeling (GMM)^10^, XGBoost^11^, and support vector machines (SVM)^12^ were used by providing the handcrafted features as inputs, and now deep learning models based state-of-the-art models are proposed. Deep neural networks (DNNs), including convolutional neural networks (CNNs) and recurrent neural networks (RNNs), demand significantly more labeled data than traditional machine learning models to achieve optimal performance. As a result, using a large and carefully selected dataset is crucial when using deep learning techniques, as the model’s performance increases with the caliber and volume of data.

To support this task, we curate FSM5, a task-specific forest acoustic dataset designed to characterize critical and ambient sound events in forest environments. The audio clips were gathered from several online resources, such as the ESC-50, Urbansound8K, and Google Audio Set databases^13^. The collection has two primary branches: ambient forest sounds and critical acoustic events. The ambient sounds of the forest are further subdivided into animal sounds and natural sounds, which include a range of everyday noises such as insects, water pouring, thunderstorms, chickens, sheep, roosters, and frogs. The category of critical sounds encompasses noises such as firearm discharges, woodworking, and diverse automobile sounds.

Existing works have explored pre-trained models such as ResNet^14^, DenseNet^15^, SqueezeNet^16^, AlexNet^17^, VGG^18^, and MobileNetV1^19^ leveraging transfer learning to adapt these models for specific audio classification domains. Also, models like SoundCLR^20^, which uses a modified ResNet architecture for the classification task, achieve high accuracy across different SER datasets, and few of the works emphasize the importance of pre-trained weights from the ImageNet model in improving accuracy, particularly in the ESResNet^21^. While transfer learning offers practical advantages in deploying DL models, it also poses challenges related to domain mismatch and dataset biases, underscoring the need for careful consideration and precautionary measures in ESR systems.

In this work, we propose ForNet, a two-stage framework for sound event classification in forest environments. The core motivation behind the two-stage design is to decouple representation learning from decision making: CNNs are used to learn discriminative and separable audio embeddings from handcrafted acoustic features, while ensemble classifiers exploit these embeddings to construct more robust decision boundaries, particularly under limited and heterogeneous data conditions. This design choice is empirically validated through consistent performance gains over end-to-end CNN classifiers across datasets. The key highlights of our work are summarized below.

- We propose ForNet, a two-stage sound event classification framework in which CNNs learn discriminative audio embeddings and ensemble classifiers leverage these representations to improve classification robustness under limited data.
- We curate FSM5, a task-specific forest sound dataset assembled from publicly available sources, designed to support acoustic-based forest monitoring and surveillance research.
- We systematically analyze the effectiveness of MFCC, Mel, and Log-Mel representations, demonstrating that CNN-extracted embeddings combined with ensemble classifiers achieve consistent 3-7% performance gains over end-to-end CNN models.
- We provide qualitative visualization and analysis of learned audio embeddings to offer insights into class separability and to justify the effectiveness of the proposed two-stage design.
- We validate the generalizability of the proposed framework through extensive evaluation on both the FSM5 dataset and the UrbanSound8K benchmark.

## Background study : existing methodologies for ESR

The review covers a broad range of subjects, including the discussions on various hand-crafted features, machine learning and deep learning methods, specific preprocessing techniques designed for acoustic environmental monitoring, and the use of pre-trained and hybrid models for sound event recognition. This study intends to consolidate findings from many studies to provide a comprehensive understanding of the latest methodologies in soundscape analysis. Several studies^22–25^ highlight the pivotal role of MFCC in diverse audio processing applications. Ali et al.’s^22^ examination highlights MFCC’s superiority over Linear Predictive Coding (LPC) in speech recognition, emphasizing its efficacy in handling multiple speakers and various languages and mitigating background noise challenges. Copiaco et al.^23^ study complements this by showcasing MFCC’s effectiveness in multi-channel audio classification tasks, alongside other methods like Continuous Wavelet Transform-Fourier Transform (CWTFT) scalograms and Log-Mel spectrograms, underscoring the significance of feature selection and dataset balancing. Banuroopa et al.’s^24^ work further extends MFCC’s utility, illustrating its role in generating robust audio fingerprints for identification, utilizing MFCC spectrograms to create unique binary images resilient to transformations and noise. Together, these studies collectively reinforce MFCC’s versatility and reliability in enhancing audio processing tasks across different domains, from speech recognition to audio classification and identification.

Significant improvements in automatic sound detection have been reported, particularly in the areas of biodiversity monitoring, urban sound classification, and environmental sound classification (ESC). Chalmers et al.^26^ designed a technique that combines machine learning and deep learning techniques to categorize bird species based on their auditory signals, reaching noteworthy accuracy rates. Krishan Das et al.^1^ illustrate the superiority of LSTMs in urban sound categorization, exhibiting their efficacy in delivering state-of-the-art performance on datasets like UrbanSound8k. Guzhov et al.^27^ on the other hand, propose a novel method to ESC utilizing techniques from the image domain, resulting in significant gains in classification accuracy, particularly when leveraging various channels in the input signal and making significant contributions with attention mechanisms. Furthermore, Huang’s^2^ work provides promising progress in urban sound event classification through the establishment of a unique 2-order dense convolutional network, showing enhanced accuracy and adaptability compared to existing models. Finally, Segarceanu et al.^28^ analyze the application of feedforward neural networks (FFNNs) and CNNs for automatic environmental sound detection, showing the efficacy of FFNNs with specific initialization and classification procedures.

The pursuit of efficient and accurate sound event identification on resource-constrained IoT devices has prompted varied techniques across the research community. Cerutti et al.’s^29,30^ works combine extreme quantization and binary neural networks (BNNs) to customize sound event detection for the GAP8 microcontroller, achieving a stunning 77.9% accuracy with greatly decreased memory demands and high energy efficiency. Building upon this, Mohaimenuzzaman et al.^31^ explore a two-stage student-teacher strategy, boosting deep learning approaches for microcontrollers and solving obstacles like 8-bit quantization. Their technology, featuring a compression-friendly network and distillation strategies, provides encouraging results, with an embedded implementation obtaining 68% accuracy on Urbansound8k while conserving resources. Furthermore, Wang’s^32^ research digs into FPGA-based environmental sound detection, optimizing feature extraction and convolutional neural networks to alleviate computational limitations. With an emphasis on real-time performance and data minimization, the proposed system achieves an 88.33% detection accuracy, highlighting the viability of deploying sound recognition systems on edge computing devices. Together, these works underline a concerted effort to create efficient, accurate, and resource-aware approaches for sound event identification, giving substantial contributions towards achieving IoT-enabled applications in real-world circumstances.

In recent works, researchers have investigated significant methodologies by utilizing machine learning techniques for audio analysis in diverse domains. Marcelo et al.^33^ studied the viability of utilizing CNNs to identify Aedes aegypti mosquitoes through audio analysis from cell phones, achieving high accuracies ranging from 78.12% to 97.65% across multiple classifiers. Juncheng et al.^34^ conducted comprehensive research on Google AudioSet by comparing neural network architectures for audio tagging tasks, revealing information on the trade-offs between performance, efficiency, and optimization procedures. Loris Nanni et al.^35^ focused on ensembles of CNNs for environmental sound recognition, achieving competitive performance across benchmark datasets with accuracy up to 97%. Alessandro Andreadis^36^ developed a framework for identifying unlawful tree-cutting activities in forests utilizing ultra-low-power smart IoT sensors and CNN-based audio classification, obtaining an 85% accuracy while emphasizing the system’s potential for cost-effective forest monitoring. These articles jointly illustrate the efficacy of machine learning and CNNs in varied audio analysis applications, spanning from mosquito identification to environmental monitoring, delivering valuable insights for practitioners and scholars in the field.

Numerous studies have applied data augmentation in both audio and image domains, though it is more commonly used in image-related tasks. This strategy frequently involves utilizing a range of image augmentation techniques, including fill, rotation, flips, and modifications to brightness, among other methods. On the other hand, few approaches^37,38^ advocated by Zohaib Mushtaq et al. and Loris Nannini et al. focus on enhancing audio directly. They promote the use of audio-level augmentation techniques such as pitch shifting, noise adding, and time stretching in their work. Madhu et al.^39^, in their study, apply Generative Adversarial Networks (GANs) for data augmentation in ESC research, demonstrating the effectiveness of synthetic data generation in enhancing classification accuracy. The direct enhancement of audio data holds the potential to deliver superior advantages in the field of audio augmentation, hence creating new opportunities for upgrading audio-based tasks.

The majority of current works mainly concentrate on classifying a fixed set of known sound events using benchmark datasets like ESC-50, UrbanSound8K, and FSC22, despite notable advancements in environmental sound classification. Although various handcrafted features, deep architectures, and augmentation strategies have helped these studies achieve high classification accuracies, they frequently fail to detect anomalous or unpredictable acoustic events, which are critical in real-world forest monitoring applications. In order to close this gap, we present a novel dataset (FSM5) that goes beyond fixed taxonomies to incorporate uncommon and unusual sounds that are frequently missed in traditional datasets, such as gunshots, illicit logging, and vehicle intrusions. Furthermore, we suggest a two-stage architecture that uses a CNN to learn deep representations under supervision before classifying the data using ensemble models to detect deviations that point to critical sound events. This hybrid framework offers a workable and scalable solution for acoustic environmental monitoring in delicate ecosystems by improving performance on standard datasets while also exhibiting strong generalization on the suggested FSM5 dataset.

## Existing datasets for SER

We propose to use the existing Urbansound 8k^40^ and ESC 50^41^, and Google Audio Set databases^13^ to build the FSM5 dataset. We pick ambient and anomalous sound events concerning forest environments and design a separate database to train a classifier for detecting sound events in forest environments. The above-mentioned datasets are discussed in the following subsections.

### UrbanSound8k

UrbanSound8K^40^ is a comprehensive collection of various urban sounds, including street noises, machinery, and human activities, that were recorded in different real-life environments. It consists of a total of 8732 audio samples. Every audio clip is meticulously labeled with information such as sound category, geographic coordinates, and recording equipment, allowing researchers to investigate a wide range of urban sounds for different tasks. UrbanSound8K is a helpful resource for the development and evaluation of sound recognition algorithms in urban audio analysis and intelligent city systems. It offers a wide range of urban audio samples and rich annotations, making it an extensive and useful tool. The class labels of the dataset are as follows: air conditioner, car horn, children playing, dog bark, drilling, engine idling, gunshot, jackhammer, siren, street music.

### ESC50

ESC-50^41^ is a collection of 2,000 audio recordings that are divided into 50 classes. It includes a wide range of sounds that are commonly heard in everyday life, such as animal calls, musical instruments, and home noises. Every sound clip is carefully annotated with its relevant category, which aids in the training and assessment of machine learning models for sound classification tasks. The ESC-50 dataset facilitates study in acoustic scene analysis, automatic sound recognition, and related topics by offering a carefully selected collection of different environmental sounds that are clearly categorized.

### Google audio set

AudioSet^13^ is a comprehensive dataset featuring an evolving ontology of 632 audio event classes. It includes a collection of 2,084,320 ten-second sound clips, each annotated by human labelers and sourced from YouTube videos. The ontology is structured as a hierarchical graph of event categories, encompassing a diverse array of sounds, including human and animal noises, musical instruments and genres, as well as everyday environmental sounds. Few audio samples from the Audioset data set were used in the FSM5 dataset.

## Forest Sound scape Monitor (FSM5) dataset

**Fig. 1:**
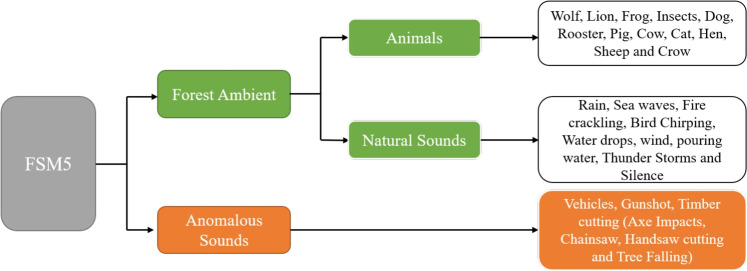
FSM5 dataset Taxonomy.

Our work introduces the Forest Sound-scape Monitor (FSM5) dataset, designed for forest surveillance using audio signals. Unlike general-purpose datasets such as UrbanSound8K and ESC-50, FSM5 focuses exclusively on forest-relevant acoustic events, with particular emphasis on distinguishing ambient forest sounds from critical human or activity-induced acoustic events. Creating such a dataset could address the limitations of existing domain-specific knowledge and improve model performance in targeted areas. The dataset is used for narrowing down the task of sound event classification to the woodland scenario. As shown in Fig. 1, the dataset is comprised of 5 major classes. The classes include animal sounds, natural sounds, chainsaw sounds, gunshots, and vehicular sounds. Animal and natural sounds are quite common in any forest area, and the other 3 groups can be considered critical acoustic events.

FSM5 comprises 2,258 audio clips organized into five balanced high-level classes: Animal, Natural Sound, Vehicle Sound, Gunshot, and Timber Cutting. as presented in Table 1. The dataset is curated from ESC-50, UrbanSound8K, the Freesound database, and Google AudioSet, unified under a consistent labeling, sampling rate, and duration format. All audio clips are resampled to 44.1 kHz, 16-bit PCM format.

**Table 1:** Audio Sample Distribution Across Classes.

Class	Number of samples
Animal	460
Natural Sound	454
Car Sound	450
Gunshot	449
Timber Cutting	445
Total	2258

**Fig. 2:**
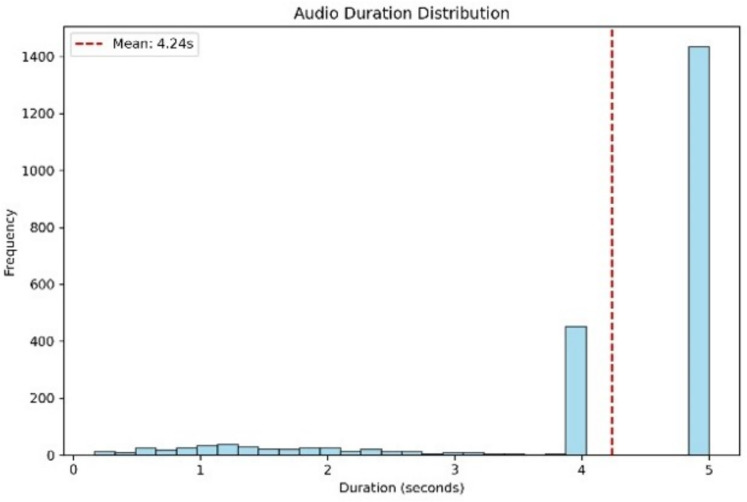
Distribution of Auido Durations.

FSM5 is divided into two wide categories: forest ambient sound and abnormal sound. The ambient category consists of an eclectic variety of animal sounds from wolf and lion to dog, rooster, pig, cow, cat, hen, frog, insects, sheep, and crow as well as environmental sounds, including rain, sea waves, fire crackling, bird chirping, water drops, wind, pouring water, thunderstorms, and silence. The critical sound events category encompasses three categories: vehicular sounds, gunshots, and timber-cutting processes. In timber cutting, finer differentiation is recorded, such as axe impacts, chainsaw cutting, handsaw cutting, tree falling, and wood cutting, which renders the dataset responsive to various kinds of logging disturbances. This taxonomy enables focused evaluation of acoustic monitoring systems that must separate normal forest soundscapes from potentially harmful or illegal activities.

**Class-wise Data Collection and Selection Crieteria**: Comprehensive dataset statistics, including source-wise contributions and clip duration characteristics, are reported in Table 3. Animal sounds class includes vocalizations and movement-related sounds of forest and domestic animals, serving as indicators of biodiversity and wildlife activity. The subclasses include wolf, lion, frog, insects, dog, rooster, pig, cow, cat, hen, sheep, and crow. Animal sound samples were primarily sourced from ESC-50 ($\approx$75%), with supplementary samples from the Freesound database ($\approx$14%) and Google AudioSet ($\approx$11%). Freesound clips were selected using keyword-based queries (e.g., wolf, forest animals, lion) and manually verified to ensure outdoor, non-overlapping recordings with minimal background noise. Vehicle sounds indicate unauthorized access or transport activity in forest regions. Vehicle category includes engine noise and vehicle movement sounds and was curated from UrbanSound8K ($\approx$20%), ESC-50 ($\approx$30%), Freesound ($\approx$10%), and Google AudioSet ($\approx$40%). Google AudioSet clips were extracted from labeled YouTube segments corresponding to vehicle-related sound events, ensuring clear temporal alignment with the annotated acoustic activity. Timber cutting class captures sounds associated with illegal logging and deforestation activities. Sub-classes include axe impacts, chainsaw cutting, handsaw cutting, and tree falling. The majority of timber-cutting samples were extracted from ESC-50 ($\approx$72%), while Freesound ($\approx$22%) was used to collect finer-grained and less represented events such as axe impacts and tree falling. A small fraction of diverse mechanical cutting sounds was obtained from Google AudioSet ($\approx$6%).

**Audio Extraction, Filtering and Normalization**: All audio clips in FSM5 were standardized to durations ranging from 1 to 5 seconds, depending on the acoustic event type, with an average clip length of 4.24 seconds, as shown in Fig. 2, with the majority of the clips ranging from 4 to 5 seconds . During curation, only recordings with clearly audible target acoustic events were retained, while indoor recordings, clips dominated by non-target background noise, and multi-event recordings containing overlapping sound sources were excluded. Audio samples from the Freesound platform were acquired using its publicly available API endpoints, enabling automated keyword-based search, metadata retrieval, licensing verification, and bulk downloading through Python scripts without manual intervention. For Google AudioSet, audio clips were extracted from labeled YouTube segments, and only those segments with strong label confidence and clear temporal alignment with the target acoustic event were selected for inclusion in the dataset.

**Dataset Bias and Duplicate Screening**: Since FSM5 is curated by combining recordings from multiple publicly available datasets, variations in recording conditions, microphone characteristics, environmental noise, and annotation standards may introduce domain-specific biases. Such heterogeneity can influence model generalization in real-world deployments, particularly in unseen acoustic environments. Despite these precautions, the dataset should be viewed as a task-specific curated benchmark rather than a fully in-the-wild forest recording collection.

To reduce potential data leakage and redundancy, a systematic duplicate and near-duplicate screening procedure was conducted across all 2,258 audio clips in FSM5. Initially, exact duplicate detection was performed using SHA-256 file hashing and filename consistency checks. No exact duplicate groups or repeated filenames were identified across the dataset. Subsequently, near-duplicate analysis was conducted using spectrogram-based audio fingerprinting and cosine similarity. Each audio clip was converted to mono, resampled to 8 kHz, normalized, and transformed into compact $64 \times 32$ log-spectrogram representation, then flattened, and L2-normalized to form an audio fingerprint for similarity comparison. Using a conservative cosine similarity threshold of 0.999, only 10 highly similar candidate pairs were identified for manual inspection, as summarized in Table 2. Importantly, all detected pairs belonged to the same class, and no cross-class duplicate or contradictory samples were observed. The identified near-duplicate candidates correspond to less than 1% of the dataset, indicating minimal redundancy while acknowledging that a limited number of acoustically similar within-class recordings may exist due to the curated nature of the dataset.

**Table 2:** Examples of highly similar audio clip pairs identified during near-duplicate screening using spectrogram-based fingerprint similarity.

Cosine similarity	File A	File B	Class
0.999993	Timber_Cutting/11_11130.wav	Timber_Cutting/11_11142.wav	Timber Cutting
0.999984	Natural_Sound/6_10669.wav	Natural_Sound/6_10670.wav	Natural Sound
0.999765	Timber_Cutting/10_11021.wav	Timber_Cutting/16_11648.wav	Timber Cutting
0.999384	Car_Sound/1766-39-4.wav	Car_Sound/1766-788-4.wav	Car Sound
0.999357	Car_Sound/1766-33-4.wav	Car_Sound/1766-655-4.wav	Car Sound
0.999244	Gunshot/1766-296-3.wav	Gunshot/1766-98-3.wav	Gunshot
0.999232	Gunshot/1766-303-3.wav	Gunshot/1766-309-3.wav	Gunshot
0.999201	Timber_Cutting/10_11056.wav	Timber_Cutting/16_11608.wav	Timber Cutting
0.999146	Car_Sound/1766-75-4.wav	Car_Sound/1766-974-4.wav	Car Sound
0.999013	Gunshot/1766-299-3.wav	Gunshot/1766-310-3.wav	Gunshot

**Table 3:** FSM5 dataset composition: source distribution and clip duration.

Class	UrbanSound8K	Freesound	ESC-50	AudioSet	Clip duration
Animal	NA	14%	75%	11%	1–5 s
Vehicle Sound	20%	10%	30%	40%	3–4 s
Gunshot	74%	16%	NA	10%	1–5 s
Natural Sound	NA	21%	59%	20%	5 s
Timber Cutting	NA	22%	72%	6%	5 s

**Fig. 3:**
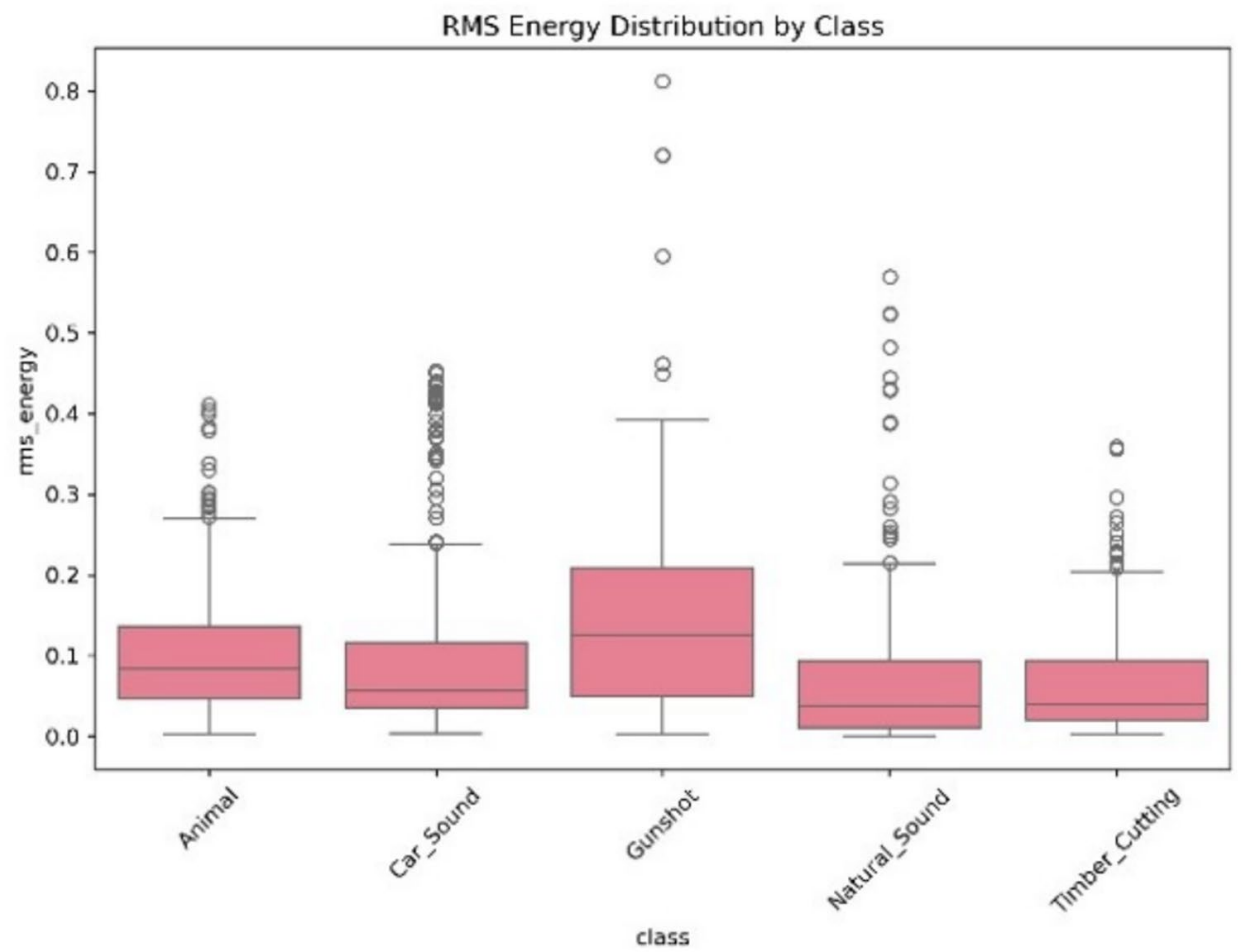
RMS Energy Distribution by Class.

**Fig. 4:**
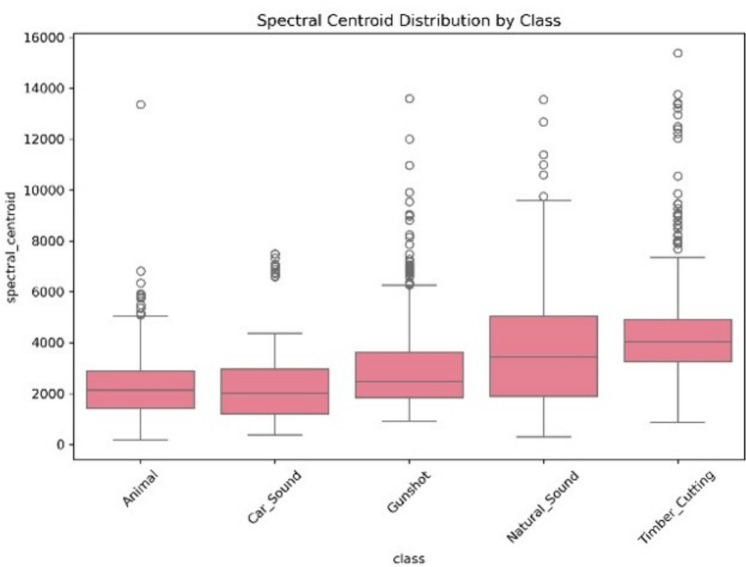
Spectral Centroid Distribution.

The acoustic feature analysis of the dataset further underlines the significance of the proposed dataset. Class balance is paid close attention to, shunning skew that might prejudice model training. RMS energy profiles presented in Fig. 3 indicate that gunshots (anomalous) are differentiated with high-energy peaks, whereas natural and animal sounds tend to be smoother and less energetic. Trends in the spectral centroid indicate that timber-cutting events move towards higher frequency bands, as opposed to the natural and animal classes, which group in the lower bands. Zero-crossing rate distributions unambiguously distinguish between transient-dense anomalous events such as timber sounds and gunshots and from the smoother ambient classes. The same trend can be also seen in Fig. 4, Where spectral rolloff and bandwidth signal that anomalous events have a wider frequency range than the narrower range of forest ambient sounds. In future work, the FSM5 dataset will be further expanded with additional relevant and more realistic forest acoustic events to better capture the diversity and complexity of real-world forest soundscapes.

## ForNet: a two-stage audio classifier

The objective of this work is to learn discriminative acoustic embeddings that effectively separate ambient forest sounds from critical forest-related acoustic events. To achieve this, we propose ForNet, a two-stage classification framework that explicitly decouples representation learning from decision making. This design choice allows the model to leverage the strengths of deep convolutional networks for feature extraction while exploiting ensemble classifiers to construct robust decision boundaries, particularly under limited and heterogeneous data conditions.

### Stage 1: learning CNN representations

**Fig. 5:**
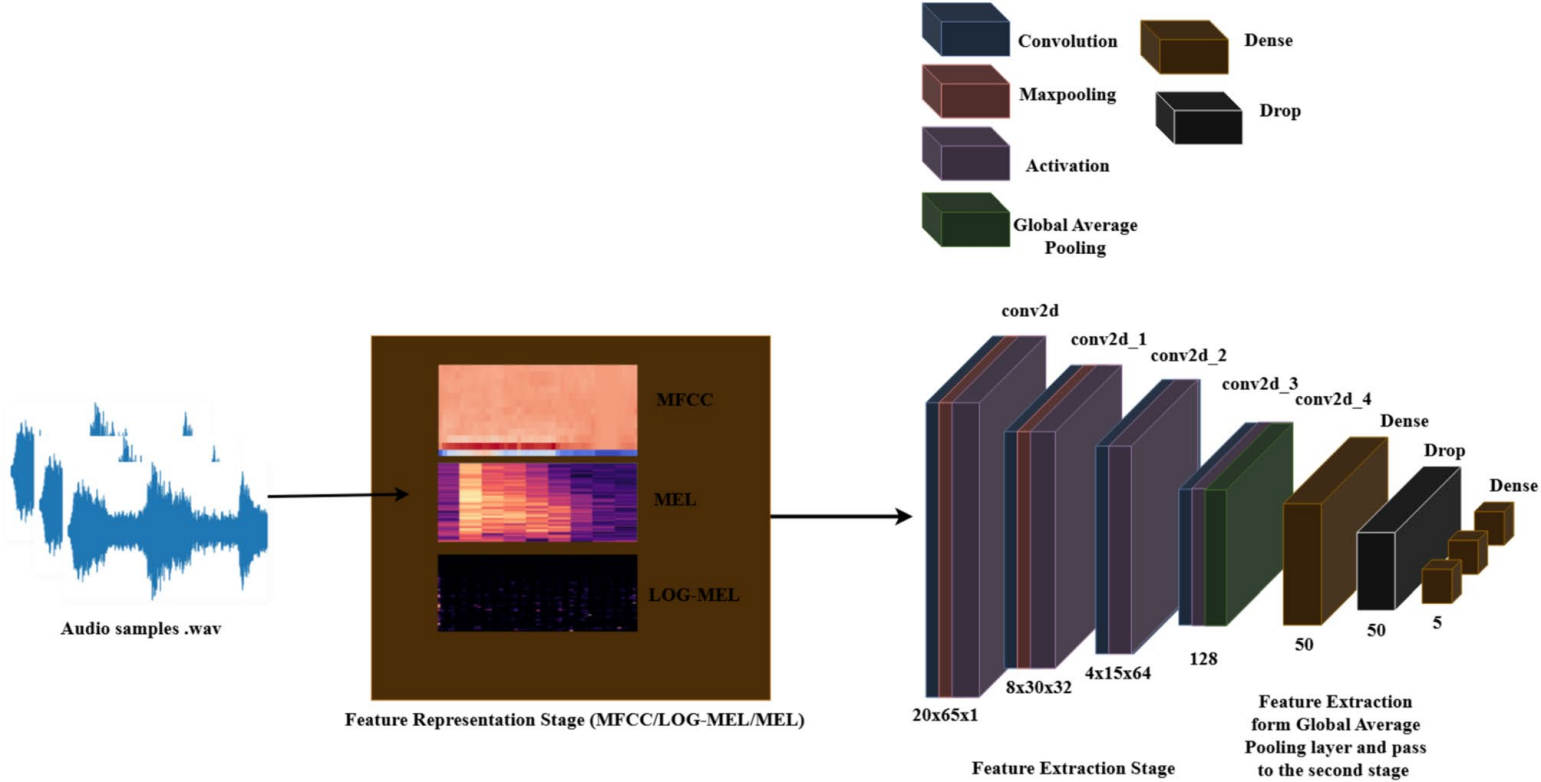
ForNet: Feature Extractor.

In the first stage, ForNet employs a CNN exclusively for representation learning. Input audio signals are transformed into time–frequency representations using MFCC, Mel, or Log-Mel features, as illustrated in Fig. 5. The corresponding input dimensions for MFCC, Mel, and Log-Mel representations are $20\times 65$, $128\times 66$, and $128\times 128$, respectively. Although the input dimensions vary across feature types, the overall CNN architecture remains unchanged. The CNN consists of only three convolutional blocks with progressively increasing filter depth (32, 64, and 128 filters) and kernel sizes of $5\times 5$, $4\times 4$, and $3\times 3$, respectively. A relatively shallow CNN architecture was intentionally adopted to balance representational capacity and computational efficiency, particularly considering the moderate scale and heterogeneous nature of the FSM5 dataset. Instead of performing direct classification through fully connected layers, Global Average Pooling (GAP) is applied after the final convolutional layer to obtain a compact 128-dimensional embedding for each audio sample. GAP was preferred over dense fully connected representations since it produces more compact and spatially aggregated embeddings, improving generalization while reducing parameter complexity. These embeddings form the input to the second-stage ensemble classifiers and serve as the primary discriminative representation used throughout the proposed framework. The learned embeddings provide a discriminative and lower-dimensional feature space that can be effectively exploited by ensemble classifiers in the second stage for improved class separation.

#### Stage 2:learning ensemble models

The learned CNN embeddings are subsequently used to train ensemble classifiers such as Random Forest and XGBoost. These classifiers operate exclusively on the GAP-layer embeddings and are responsible for the final decision-making process. The motivation behind this design is that ensemble methods can exploit the separability of CNN-learned representations to construct more robust decision boundaries than end-to-end softmax-based CNN classifiers, particularly under limited and heterogeneous data conditions. Random Forest performs classification through aggregation of multiple decision trees trained on bootstrapped subsets of data, while XGBoost iteratively refines decision boundaries using gradient-boosted trees with regularization. Their corresponding formulations are given in Eqs. (1) and (2). Empirical results demonstrate that coupling ensemble learning with CNN-derived embeddings consistently improves classification performance over standalone CNN classifiers.

1 $$\begin{aligned} C_{\text {RandomForest}} \leftarrow \text {majorityVote}\{C_i(x)\}_{1}^{n} \end{aligned}$$

where $C_i(x)$ is the $i^{\text {th}}$ random tree’s predicted classification.

The objective function of the XGBoost classifier:

2 $$\begin{aligned} L^{(t)} = \sum _{i=1}^{n} l(y_i, \hat{y}_i^{(t-1)} + f_t(x_i)) + \Omega (f_t) \end{aligned}$$

Where $L^{(t)}$ represents the objective function at iteration *t*, $l(y_i, \hat{y}_i^{(t-1)} + f_t(x_i))$ is the cost function which calculates the difference between the true value $y_i$ and the predicted value $\hat{y}_i^{(t-1)}$ from the previous iteration plus the contribution of the new decision tree $f_t$ on the input features $x_i$, and $\Omega (f_t)$ represents the regularization term.

**Experimental Control and Baseline Comparison**: To clearly isolate the effect of the proposed two-stage design, an end-to-end CNN with softmax output is trained as a baseline under identical feature representations and training conditions. In contrast, ForNet replaces the softmax classifier with ensemble models trained on GAP-layer embeddings. Empirical results consistently show that embedding-based ensemble classification outperforms end-to-end CNN classification, demonstrating that decoupling representation learning and classification is a key factor behind the observed performance gains.

## Implementation details

**Fig. 6:**
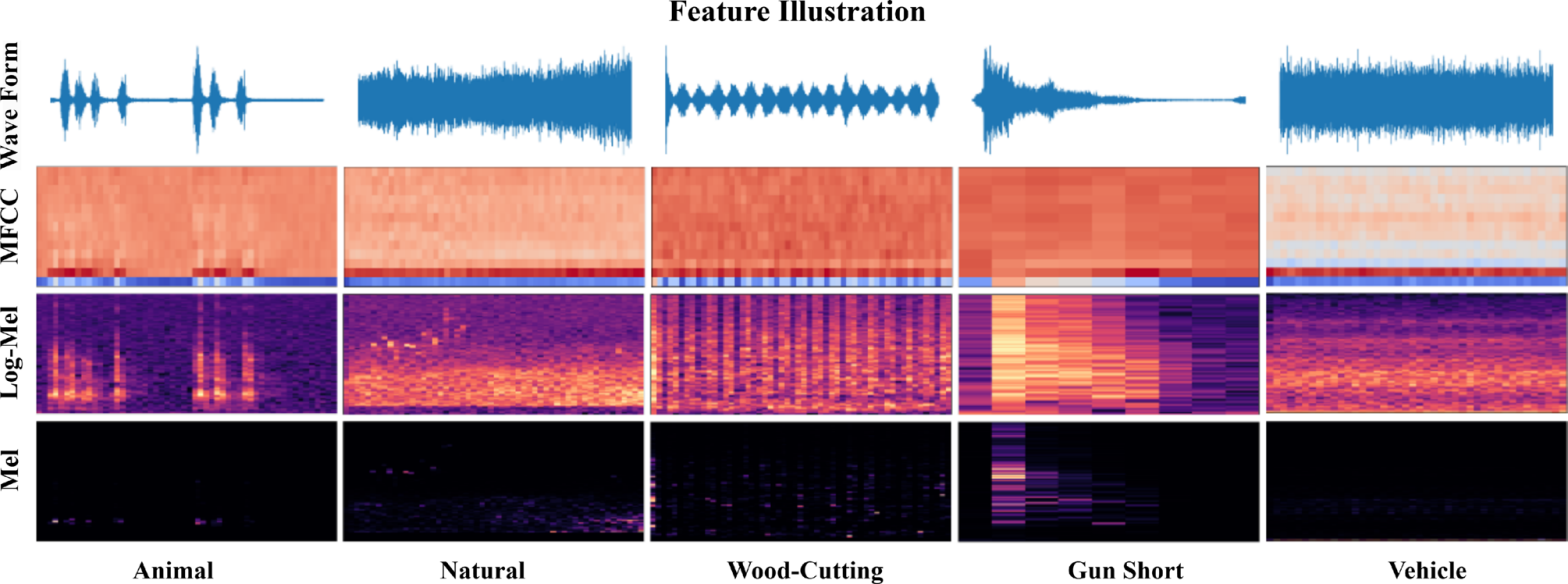
Feature Illustration of FSM5 Dataset.

**Audio Feature Extraction**: Audio signals were transformed into MFCC, Mel spectrogram, and Log-Mel spectrogram representations prior to training. Feature extraction parameters were selected empirically based on classification performance and representation stability across the FSM5 dataset. For MFCC features, the hop length and $n_{\text {fft}}$ were set to 2048. Log-Mel spectrograms were extracted using a hop length and $n_{\text {fft}}$ of 1034, while Mel spectrograms used $n_{\text {fft}}=2024$ and a hop length of 2024. The number of Mel-frequency bands ($n_{\text {mels}}$) was fixed at 128 for all Mel-based representations. During preliminary experiments, conventional window configurations such as 1024 produced comparatively lower classification stability on FSM5, particularly for transient forest acoustic events. The selected configurations provided improved time–frequency resolution and more discriminative representations for both ambient and critical acoustic events. In particular, Log-Mel representations consistently yielded stronger class separability and improved downstream ensemble performance across multiple experimental settings. The MFCC, Mel spectrogram, and Log-Mel representations are computed using Eqs. (3), (4), and (5), respectively. Figure 6 presents representative feature visualizations from FSM5 across different acoustic event categories, including ambient forest sounds and critical acoustic events. The MFCC features are represented using the power spectrum from the Short-Time Fourier Transform (STFT) as $|X(f)|^2$, with $H_m(f)$le 5’ being the Mel filterbank for the $m$-th band. The formula for MFCCs is given by:

3 $$\begin{aligned} \text {MFCC}_n = \sum _{m=1}^{M} \log \left( \sum _{f=1}^{\frac{N_{\text {FFT}}}{2}} |X(f)|^2 \cdot H_m(f) \right) \cdot \cos \left( \pi n \frac{(m - 0.5)}{M}\right) \end{aligned}$$

4 $$\begin{aligned} \text {MelSpec}(m, t) = \sum _{f=1}^{\frac{N_{\text {FFT}}}{2}} |X(f, t)|^2 \cdot H_m(f) \end{aligned}$$

where $H_m(f)$ represents the Mel filterbank for the $m$-th band, $N_{\text {FFT}}$ is the number of FFT points, and $m$ corresponds to the Mel filter index.

5 $$\begin{aligned} \text {LogMelSpec}(m, t) = \log \left( \text {MelSpec}(m, t) + \epsilon \right) \end{aligned}$$

where $\text {MelSpec}(m, t)$ is the Mel spectrogram for Mel band $m$ at time $t$, and $\epsilon$ is a small constant added to avoid undefined values when the spectrogram value is zero.

Figure 6 illustrates the features of the FSM5 Dataset, i.e., the MFCC, log-mel spectrogram, mel-spectrogram, and their accompanying waveforms. Starting from the left, the first common source of animal audio, the second column represents the audio of natural sound. The third depicts the wood-cutting activity, while the fourth and final indicate the gun-shot and vehicular movements respectively.

**Training and Optimization**: During training, the model is optimized using the Adam optimizer with categorical cross-entropy as the loss function. The model’s performance is evaluated based on accuracy, and early stopping with patience of 10 epochs is implemented. To prevent overfitting, a dropout layer with a dropout rate of 50% is added after the first dense layer. Table 4 summarizes the hyperparameters used for all ensemble and classical classifiers in the ForNet framework. Default settings were retained where appropriate to ensure stability and fair comparison, while key parameters were explicitly fixed to enable reproducibility across datasets and experimental runs.

**Table 4:** Hyperparameters of ensemble and classical classifiers used in the ForNet framework.

Classifier	Hyperparameter	Value used
Random Forest (RF)	Number of trees (n_estimators)	100
	Criterion	Gini
	Max depth	None
	Min samples split	2
	Min samples leaf	1
	Feature selection	Auto
	Bootstrap	True
	Random state	42
Gradient Boosting (GB)	Number of estimators (n_estimators)	100
	Learning rate	0.1
	Max depth	3
	Loss function	Deviance
	Subsample	1.0
	Random state	42
XGBoost (XGB)	Number of estimators (n_estimators)	100
	Learning rate (eta)	0.3
	Max depth	6
	Objective	Multi-class softmax
	Evaluation metric	Log-loss
	Subsample	1.0
	Column sample	1.0
	Random state	42
Support Vector Machine (SVM)	Kernel	RBF
	Regularization (C)	1.0
	Gamma	Scale
	Probability estimation	Enabled
	Random state	42
Logistic Regression (LR)	Solver	LBFGS
	Max iterations	1000
	Regularization	L2
	Multi-class handling	Auto
	Random state	42

**Global settings:** StandardScaler normalization; 10-fold stratified cross-validation;

80/20 stratified train–test split; Accuracy, Precision, Recall, F1-score metrics;

Friedman and Wilcoxon signed-rank tests with 95% confidence intervals.

## Results

The proposed approach is assessed based on evaluation criteria such as accuracy, F1-score, precision, and recall to determine their performance. The evaluation procedure yields valuable insights into the effectiveness of the models and facilitates the choice of the most suitable technique for detecting the anomalous sound. For UrbanSound8K, we strictly adhered to the original 10-fold cross-validation protocol provided with the dataset. No fold reshuffling or reassignment was performed, ensuring consistency with standard benchmark evaluation practices. Given the near-uniform class distribution, FSM5 was split using a natural 80–20 train–test partition, ensuring balanced representation of all five classes in both subsets. For 10-fold cross-validation experiments, stratified folds were used to preserve class balance. Three distinct CNN models are trained using MFCC, mel spectrograms, and log-mel spectrograms as inputs, respectively. The subsequent sections provide a detailed performance analysis of these models on UrbanSound8k and FSM5 datasets using two approaches: the baseline CNN (End-to-End CNN) and the proposed ForNet two-stage classifier. The next section clearly discusses the performance of the baseline end-to-end CNN with softmax classifier and traditional models on the UrbanSound8K dataset and the proposed FSM5 dataset.

## Performance of traditional models and baseline CNN on Urbansound8k 8K and FSM5 dataset

**Fig. 7:**
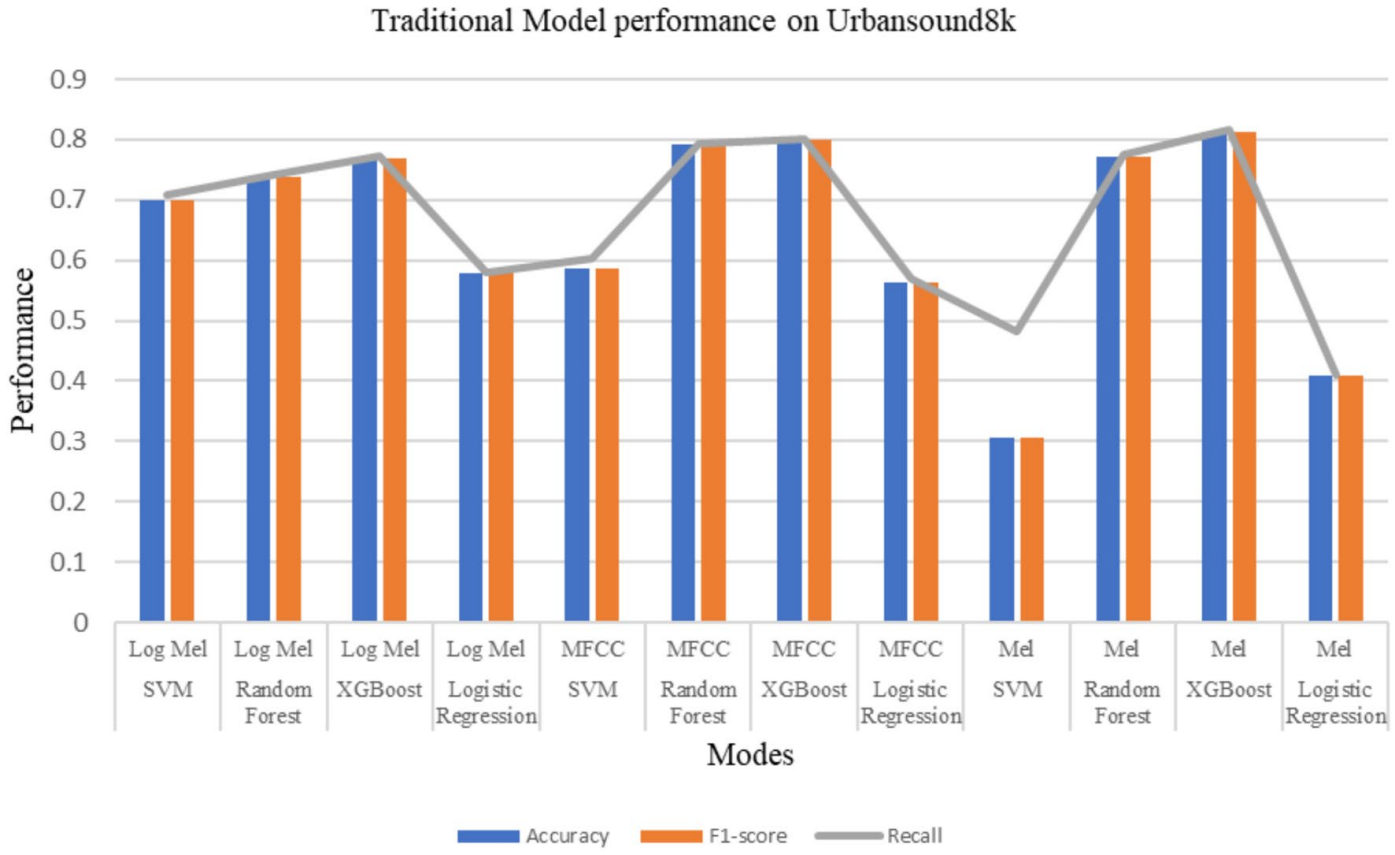
Results of Traditional Model on Urbansoun8k.

**Performance of traditional models and Baseline CNN on Urbansound8k Dataset**: Firstly, we analyze the outcomes of the performance of traditional models such as SVM, Random Forest, XGBoost, and Logistic Regression on the Urbansound8k dataset. Figure 7 explains the essential importance played by feature selection in determining model performance. Notably, Log Mel and MFCC features consistently outperform the Mel features across all classifiers, indicating their efficiency in capturing discriminative information essential for classification tasks. Among the models trained on Log Mel features, XGBoost displays the highest accuracy, F1 score, and recall, indicating its supremacy in utilizing this feature representation.

Figure 7 summarizes the performance of traditional classifiers on UrbanSound8K using MFCC, Mel, and Log-Mel representations. Across most classifiers, MFCC and Log-Mel features consistently outperform Mel representations, indicating stronger discriminative capability for environmental sound classification. Ensemble methods, particularly XGBoost and Random Forest, achieve the most stable performance across feature types, while Mel-based representations exhibit comparatively lower classification accuracy. Table 5 reports the baseline CNN performance across different feature representations. MFCC features achieve the strongest overall baseline performance on UrbanSound8K, followed by Log-Mel representations, while Mel spectrograms consistently yield lower accuracy and F1-scores.

**Table 5:** Performance Results of Baseline CNN Classifier Vs ForNet on on UrbanSound8k (Bold Indicates Maximum Performance).

Model	Feature	Accuracy (1 Fold)	10-Fold accuracy	F1-score	Recall
End-To-End CNN	MFCC	0.90	0.87	0.87	0.87
	Mel	0.78	0.75	0.78	0.77
	Log-Mel	0.87	0.83	0.87	0.87
	MFCC	0.92	**0.94**	0.92	0.92
ForNet(RF)	Mel	0.84	0.84	0.84	0.84
	Log-Mel	0.90	0.91	0.89	0.89
	MFCC	**0.94**	0.93	**0.93**	**0.93**
ForNet(XGBoost)	Mel	0.86	0.86	0.86	0.86
	Log-Mel	0.90	0.89	0.89	0.89

Bold values indicate the highest result for the corresponding evaluation metric.

**Fig. 8:**
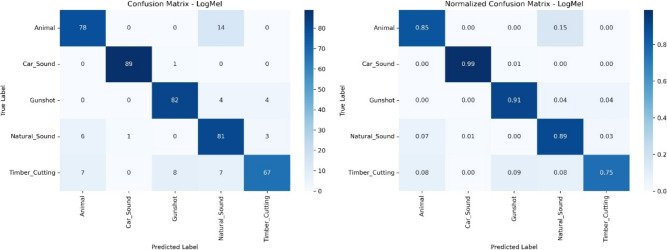
Confusion Matrix (one of the folds) Log-Mel Feature as input Baseline - CNN on FSM5 dataset.

**Fig. 9:**
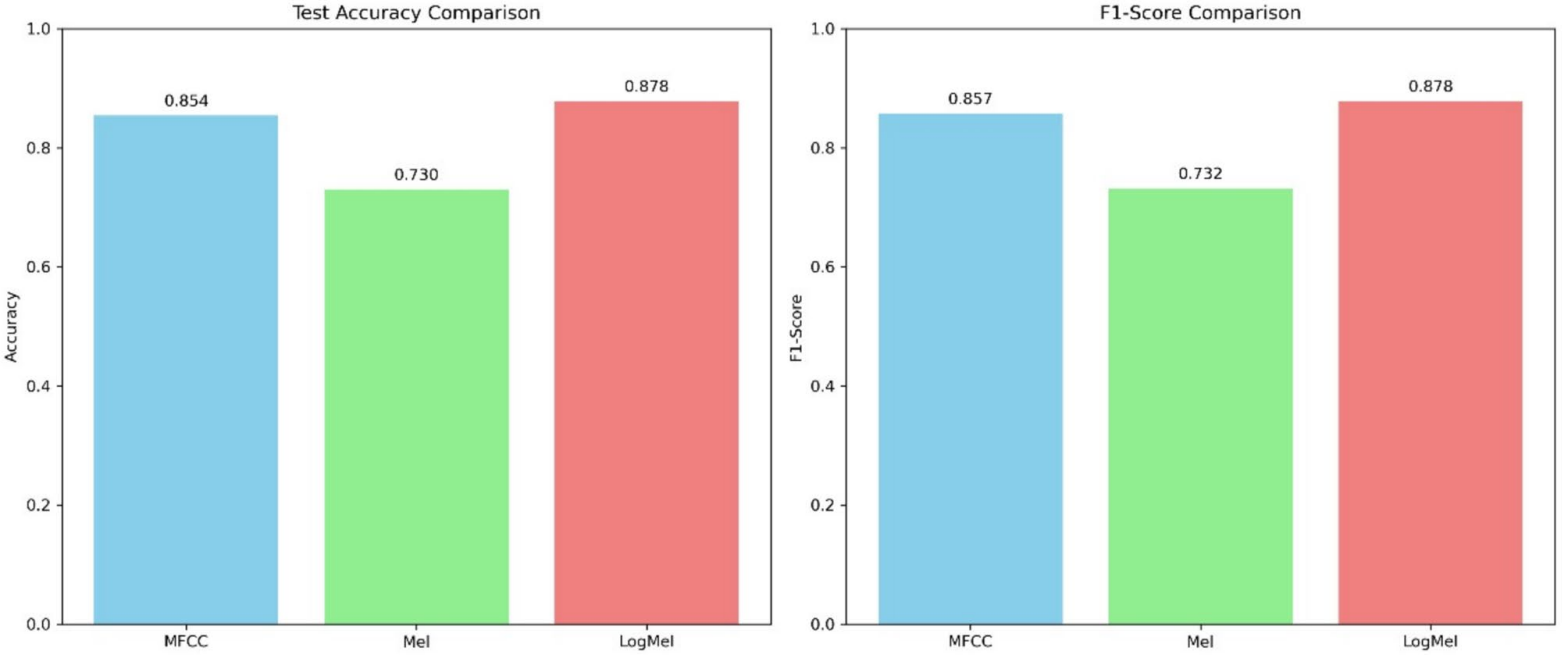
Accuracy and F1-Score of Baseline - CNN on FSM5 dataset.

**Fig. 10:**
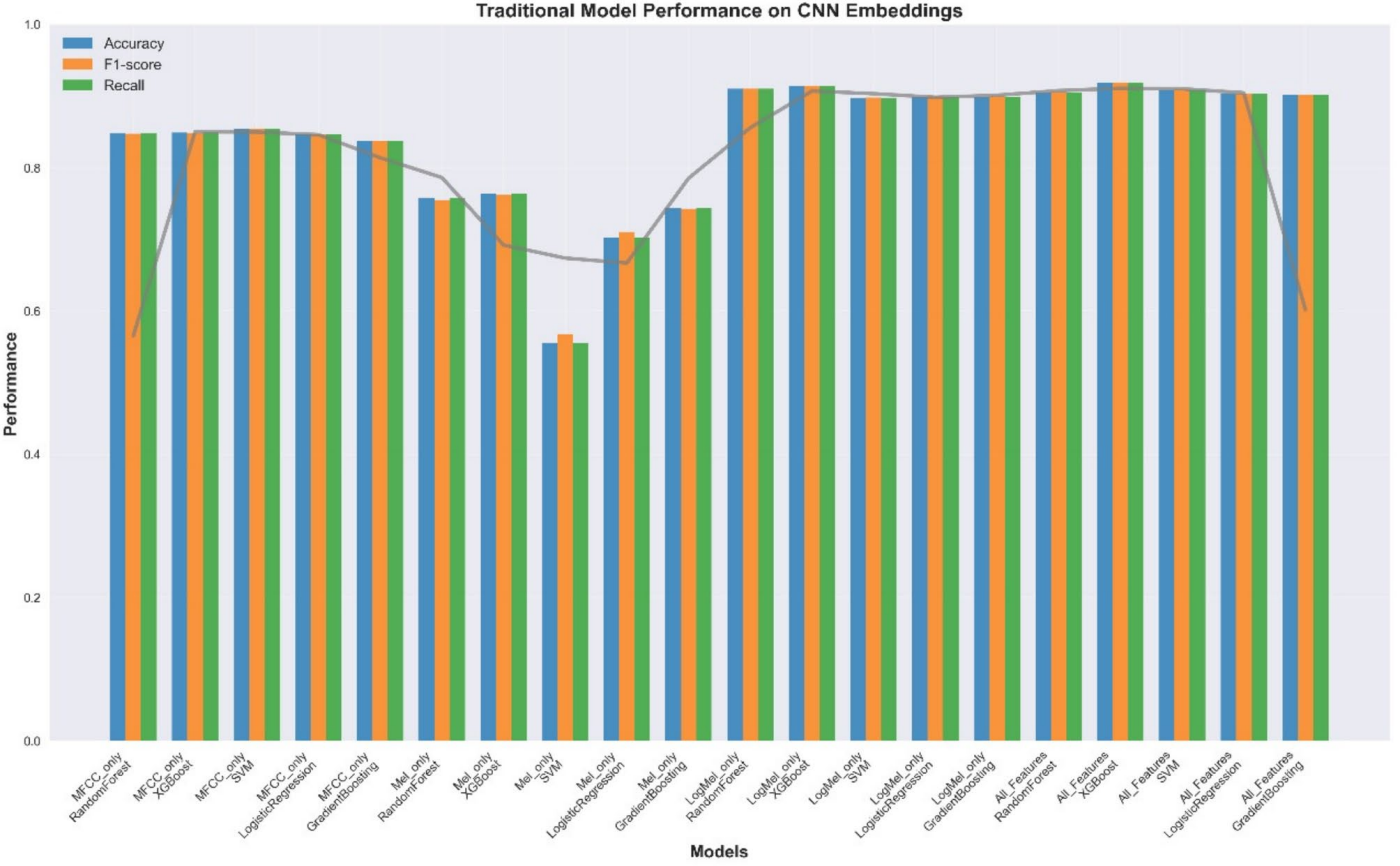
Results of Traditional Models on FSM5.

**Performance of the Baseline CNN and Traditional Approaches on the FSM5 Dataset**: The CNN model was experimented with three types of audio feature representations: MFCC, Mel spectrogram, and Log-Mel spectrogram on the FSM5 dataset. As can be seen in Fig. 9, the LogMel features gives the best overall performance with a test accuracy of 0.878 and F1-score of 0.878, outperforming MFCC with an accuracy of 0.854 and F1-score of 0.857) and Mel spectrogram with an accuracy 0.730 and F1-score of 0.732). Further performance information for the classes is presented in Fig. 8, which shows both the raw and normalized confusion matrices for the LogMel-based CNN on FSM5. The model achieves strong recognition performance for distinct acoustic events such as Car Sound, while moderate confusion is observed between acoustically similar ambient classes such as Animal and Natural Sound. Similar trends are reflected in the traditional classifier results shown in Fig. 10, where Log-Mel representations consistently provide stronger classification performance than Mel features across most classifiers.

## Performance of proposed ForNet on UrbanSound8k and FSM5 dataset

**Fig. 11:**
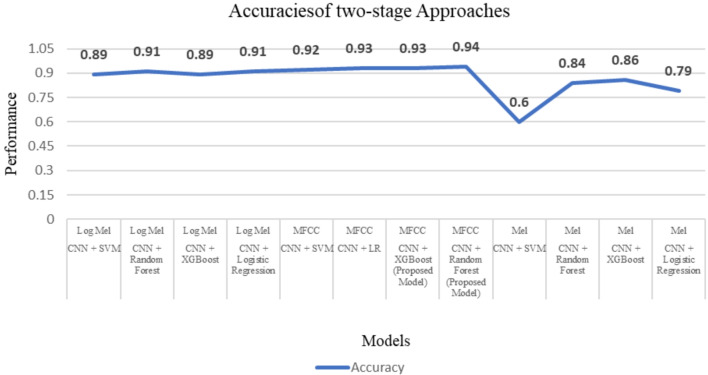
Performance Comparision of ForNet with baselines on Urbansoun8k (10-Fold).

Instead of directly feeding the handcrafted audio features to traditional models, the MFCC, Mel, and Log Mel features are fed to their respective trained CNNs. Subsequently, features are recovered from the last layer of the CNN, specifically at the global average pooling layer, as specified in the design. The input dimensions for MFCC, Mel, and Log Mel features were $20\times 65$, $128\times 66$, and $128\times 128$, respectively. These feature vectors are compressed to $1 \times 128$ vectors in the Global Average Pooling layer. These extracted audio embeddings are further trained using the ensemble models and classification is performed at the final stage. The results of this approach over the urbansound8k dataset are discussed further.

**Fig. 12:**
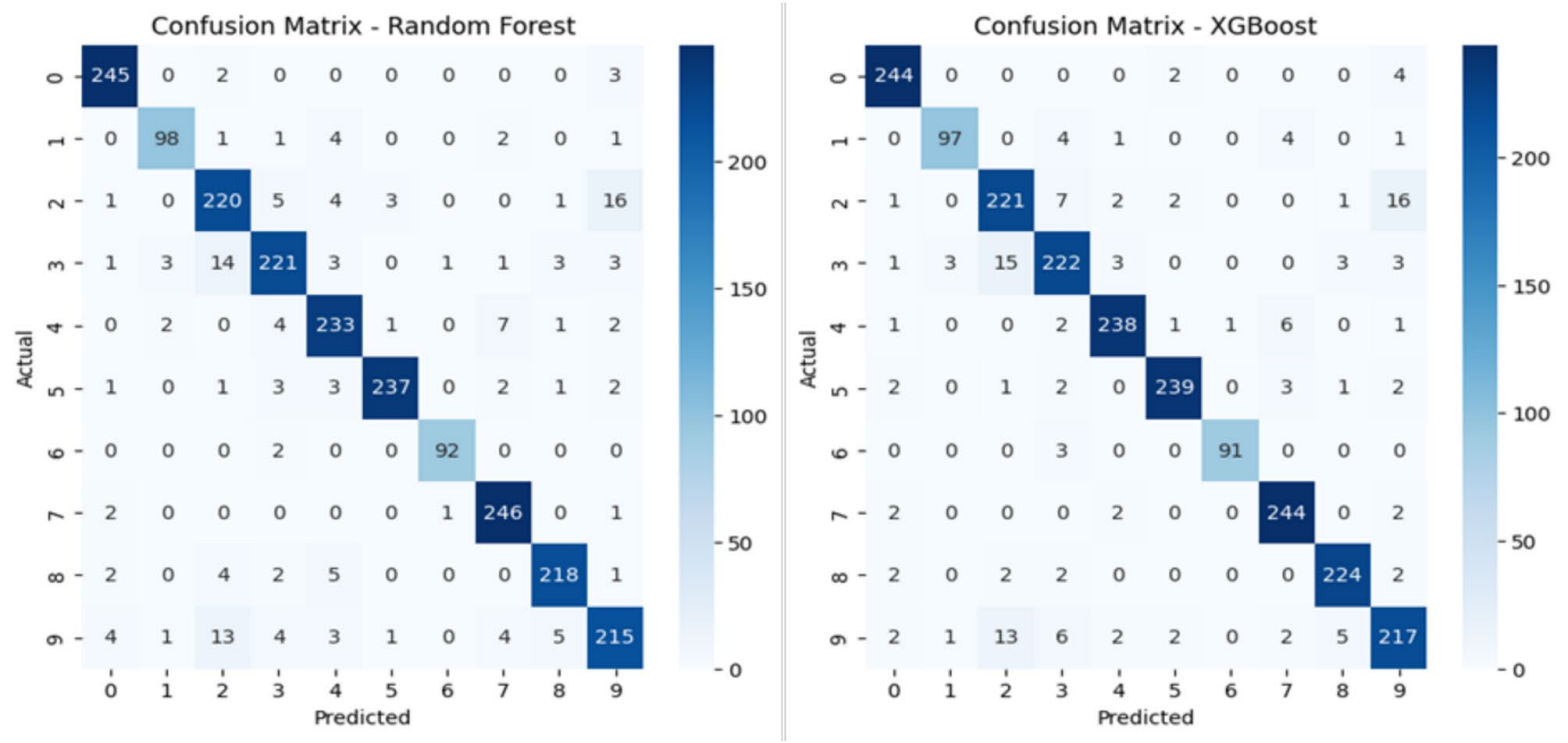
Confusion matrices of the Proposed Framework on a representative fold of UrbanSound8K.

**Performance of ForNet on Urbansound8k**: The audio embeddings extracted from the respective CNN models are used to fit various traditional classifiers. Figure 11 represents the outcomes of the two-stage ForNet framework on UrbanSound8K. The embedding-based ensemble classifiers consistently outperform the baseline CNN across all feature representations, with Random Forest and XGBoost achieving the strongest overall performance. The improvement demonstrates the effectiveness of combining CNN-derived embeddings with ensemble-based decision learning.The confusion matrices obtained using the proposed ForNet framework on a representative fold of the UrbanSound8K dataset, employing Random Forest and XGBoost classifiers, are shown in Fig. 12. Details of the corresponding labels are provided in the dataset section.

**Performance of ForNet on FSM5**:

**Fig. 13:**
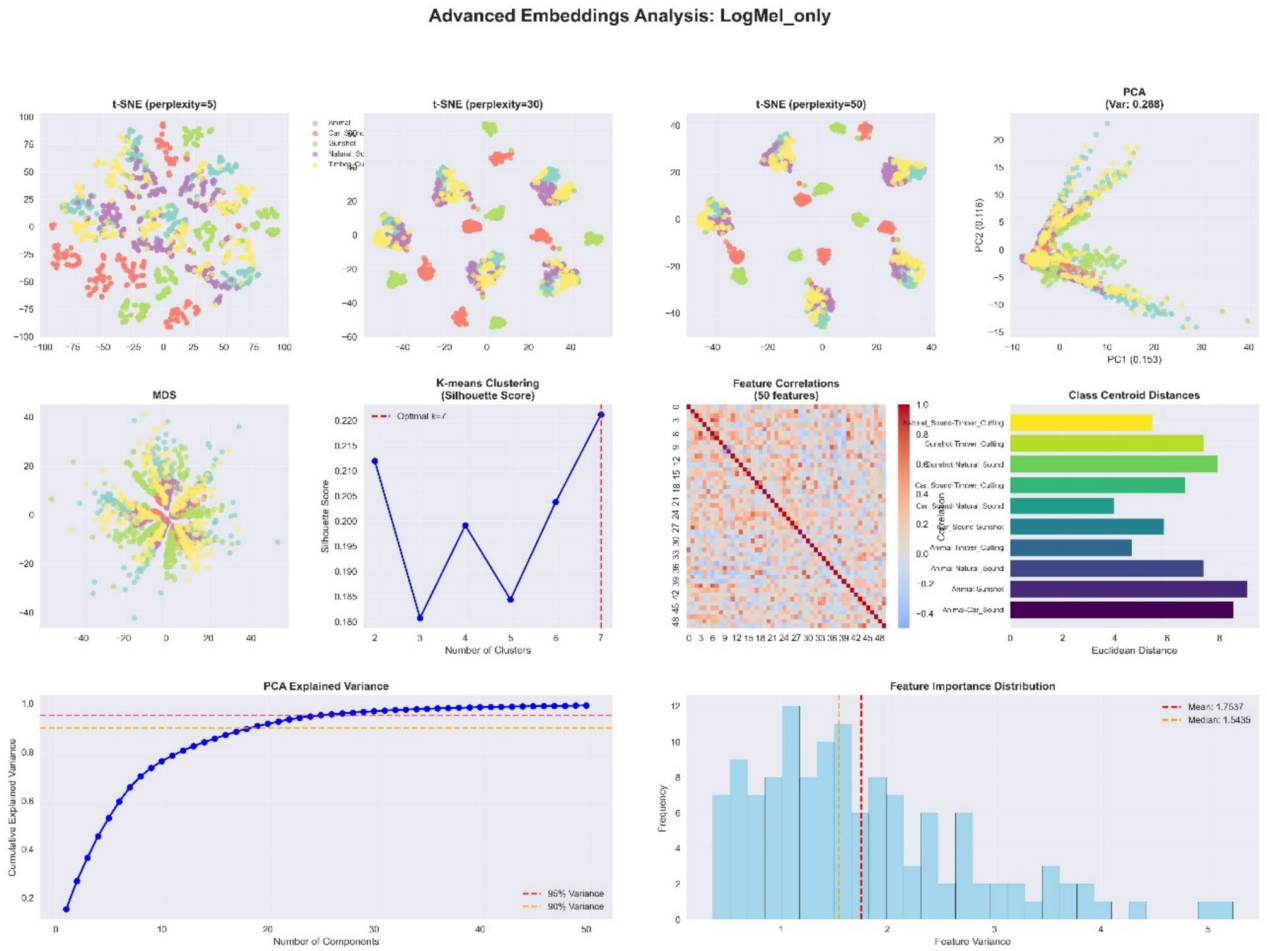
Log Mel Advanced Analysis of Representations.

For a better understanding of the representational power of the learned features via the proposed approach, deep audio embeddings were derived from CNN’s global average pooling (GAP) layer for all three input features, with Fig. 13 representing the feature analysis part with respect to LogMel features. Dimensionality reduction with t-SNE for a range of perplexity values (Fig. 13, column 1:first three plots) and PCA revealed LogMel embeddings to be tight, well-separated clusters sitting extremely close to actual class borders. The PCA explained variance curve Fig. 13, column 1:4th plot) also indicates that over 90% of the embedding variance is explained by just 20 principal components, demonstrating the compactness and expressiveness of the feature set. The K-means silhouette analysis resulted in an optimal number of clusters in line with the known number of classes and a silhouette score peak of around 0.22.

Feature correlation and variance distribution analyses (bottom row) show that the embeddings are not mostly redundant, but also heterogeneous, with a mean feature variance of about 1.75 and a median of 1.54. Class centroid distances report greater distances between some class pairs (e.g., Car Sound and Timber Cutting), as expected based on the confusion matrix results. Together, these analyses validate CNN-extracted LogMel embeddings to be highly effective and can be used for further training through the pipeline proposed, making it an effective classifier on the customy curated acoustic dataset - FSM5. In ablation study that compares LogMel-only embeddings with all-feature fusion, it is observed that combining multiple feature representations provides marginal but consistent improvements over standalone Log-Mel embeddings across ensemble classifiers.

**Table 6:** Comparison of Accuracy, Precision, Recall, and F1-Score of different approaches (with LogMel Feature) on FSM5 dataset (All results recorded for 10-Fold evaluation).

Approach (Feature used-LogMel)	Accuracy (%)	Precision (%)	Recall (%)	F1-Score (%)
Baseline CNN	$83.8 \pm 0.49$	$84.29 \pm 0.47$	$83.8 \pm 0.49$	$83.8 \pm 0.50$
ForNet (XGBoost on CNN embeddings)	${\textbf {91.4}} \pm {\textbf {1.92}}$	${\textbf {92.10}} \pm {\textbf {1.79}}$	${\textbf {91.81}} \pm {\textbf {1.82}}$	${\textbf {91.5}} \pm {\textbf {1.78}}$
ForNet (Random Forest on CNN embeddings)	$91.0 \pm 1.82$	$91.60 \pm 1.79$	$91.30 \pm 1.82$	$91.0 \pm 1.82$
ForNet (Gradient Boosting on CNN embeddings)	$89.9 \pm 1.98$	$90.41 \pm 1.79$	$89.97 \pm 1.98$	$89.9 \pm 1.96$
ForNet (SVM on CNN embeddings)	$88.7 \pm 2.65$	$90.80 \pm 2.32$	$90.25 \pm 2.65$	$88.7 \pm 2.63$
ForNet (Logistic Regression on CNN embeddings)	$89.9 \pm 1.87$	$91.35 \pm 1.83$	$91.11 \pm 1.87$	$89.9 \pm 1.86$

Bold values indicate the highest result for the corresponding evaluation metric.

The embeddings extracted from the CNN trained on Log-Mel representations were subsequently used to train multiple ensemble and classical classifiers. The comparative quantitative performance across all evaluated models is summarized in Table 6. Among the evaluated approaches, the proposed ForNet framework using XGBoost achieved the best overall performance, obtaining 91.4% accuracy, 92.10% precision, 91.81% recall, and 91.5% F1-score. Random Forest achieved closely comparable results with 91.0% accuracy and 91.0% F1-score, while Gradient Boosting also maintained competitive performance near 89.9%. In contrast, the baseline CNN using Log-Mel features achieved 83.8% accuracy and F1-score, indicating that ensemble learning over CNN-derived embeddings provides an improvement of approximately 7–8% across the primary evaluation metrics. These gains are particularly significant given the multi-class nature of FSM5 and the acoustic similarity between several forest sound categories.

Class-wise analysis further highlights the effectiveness of the proposed embedding-based framework. XGBoost achieved high recall across all classes, including Animal (89.6%), Car Sound (97.6%), Gunshot (92.4%), Natural Sound (88.8%), and Timber Cutting (88.8%), while Random Forest slightly improved Animal recall to 89.9% and achieved the highest Car Sound recall of 98.2%. Compared to ensemble approaches, linear classifiers such as SVM and Logistic Regression exhibited lower recall for acoustically overlapping classes, particularly between Animal and Natural Sound categories. The normalized confusion matrices similarly show clearer diagonal dominance for ensemble methods, indicating improved class separability and reduced misclassification. Overall, the results suggest that the CNN-derived Log-Mel embeddings form a compact and discriminative feature space that can be more effectively exploited by ensemble classifiers than standalone CNN softmax classification, leading to more robust and balanced performance across the FSM5 dataset.

## Comparison with end-to-end transformer-based audio models

To strengthen the comparison with modern transformer-based audio architectures, we additionally evaluate Wav2Vec2 ^42^ on the FSM5 dataset using the same 10-fold Stratified Cross-Validation protocol employed for ForNet. This ensures a fair and reproducible comparison under identical data partitioning conditions. The Wav2Vec2 implementation uses the pre-trained facebook/wav2vec2-base backbone with frozen transformer weights and a lightweight 128-dimensional projection head for classification. The model was trained for 50 epochs using the Adam optimizer with a learning rate of $1\times 10^{-4}$, dropout of 0.3, and cross-entropy loss. Mean pooling over transformer hidden states was used to obtain utterance-level representations. All audio clips were resampled to 16 kHz and standardized to 5 seconds. The same Stratified-K Fold configuration ($k=10$, shuffle=True, random_state=42) used for ForNet was preserved for consistency and reproducibility.

**Table 7:** 10-fold cross-validation performance comparison between Wav2Vec2 and the proposed ForNet framework on FSM5.

Model	Accuracy (%)	Precision	Recall	F1-score
Wav2Vec2	$69.62 \pm 2.24$	$75.77 \pm 2.61$	$69.57 \pm 2.25$	$69.15 \pm 2.24$
ForNet (XGBoost with Log Mel Feature)	${\textbf {91.4}} \pm {\textbf {1.92}}$	${\textbf {92.10}} \pm {\textbf {1.79}}$	${\textbf {91.81}} \pm {\textbf {1.82}}$	${\textbf {91.5}} \pm {\textbf {1.78}}$
ForNet (Random Forest with Log Mel Feature)	$91.0 \pm 1.82$	$91.60 \pm 1.79$	$91.30 \pm 1.82$	$91.0 \pm 1.82$

Bold values indicate the highest result for the corresponding evaluation metric.

**Table 8:** Class-wise performance of Wav2Vec2 on FSM5 under 10-fold Stratified Cross-Validation.

Class	Precision	Recall	F1-Score
Animal	0.8585	0.7652	0.8092
Car_Sound	0.8247	0.3556	0.4969
Gunshot	0.7598	0.8241	0.7906
Natural_Sound	0.4788	0.8458	0.6115
Timber_Cutting	0.8384	0.6876	0.7556
Macro Average	0.7520	0.6957	0.6927

The updated 10-fold cross-validation results in Table 7 show improved performance for Wav2Vec2 after extending the training duration to 50 epochs. Wav2Vec2 achieves a mean accuracy of 69.62% with a macro F1-score of 69.15%. Nevertheless, the proposed ForNet framework continues to significantly outperform Wav2Vec2, consistently exceeding 91% accuracy on the FSM5 dataset under the same evaluation protocol. The class-wise analysis in Table 8 indicates that Wav2Vec2 performs comparatively better on impulsive acoustic events such as Gunshot and Animal sounds, achieving F1-scores of 0.7906 and 0.8092, respectively. However, the model struggles considerably with acoustically heterogeneous classes such as Car Sound and Natural Sound, where overlapping environmental textures and recording variability reduce class separability. In contrast, the proposed ForNet framework demonstrates stronger robustness across all classes due to its use of domain-guided time-frequency representations. The performance gap highlights the effectiveness of combining handcrafted acoustic representations with CNN-based embedding extraction in moderate-scale environmental sound datasets. While Wav2Vec2 learns representations directly from raw waveforms, ForNet benefits from MFCC and Log-Mel representations that explicitly preserve discriminative spectral characteristics relevant to forest acoustic monitoring. Furthermore, transformer-based architectures generally require substantially larger datasets and computational resources to fully exploit their representational capacity, whereas the comparatively lightweight CNN-based ForNet architecture remains computationally efficient and better suited for deployment-oriented forest surveillance systems.

### Computational efficiency and inference cost

**Fig. 14:**
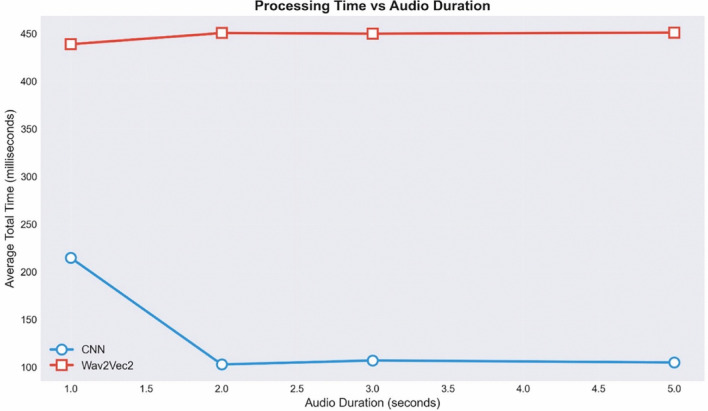
ForNet vs Wav2Vec Feature Extraction time.

Beyond accuracy, computational efficiency is a key requirement for real-world forest monitoring systems. Table 9 reports the average feature extraction time as a function of audio duration for ForNet-based inference and Wav2Vec. The reported feature extraction and inference results are based exclusively on CPU-based execution, without GPU acceleration. The plot in Fig. 14 shows that Wav2Vec2 incurs consistently high and nearly duration-invariant processing time, remaining around 440–450 ms across all audio lengths due to its fixed transformer depth and self-attention operations. In contrast, the ForNet-based approach scales more efficiently, stabilizing near 100–110 ms for longer clips, highlighting its suitability for time-sensitive deployment scenarios. The CNN based ForNet model achieves an average processing time of approximately 132 ms, whereas Wav2Vec2 requires around 447 ms per audio clip, making the CNN-based approach roughly 3.3$\times$ faster.

**Table 9:** Average processing time (ms) versus audio duration on FSM5.

Audio Duration (s)	CNN Time (ms)	Wav2Vec2 Time (ms)
1	214.5	438.6
2	102.7	450.3
3	106.8	449.6
5	104.8	450.8

## Conclusion

In conclusion, this work presents a comprehensive study on forest acoustic event classification through the introduction of the FSM5 dataset and the proposed two-stage ForNet framework. FSM5 is curated to represent forest-relevant acoustic scenarios, enabling focused evaluation of systems that must distinguish ambient forest sounds from critical human- or activity-induced events. Extensive experiments on both FSM5 and UrbanSound8K demonstrate that the proposed approach achieves strong and consistent performance across datasets. A systematic evaluation of acoustic feature representations shows that MFCC and Log-Mel features consistently yield superior discriminative capability when combined with CNN-based representation learning. By decoupling representation learning from classification, ForNet effectively integrates the expressive power of deep CNNs with the robust decision boundaries of ensemble classifiers. Among the evaluated models, XGBoost and Random Forest-based classifiers exhibit strong generalization when applied to CNN-extracted embeddings. To support reproducibility, the hyperparameters of all ensemble and classical classifiers are explicitly documented and discussed. Comparative analysis with transformer-based architectures such as Wav2Vec2^42^ further highlights the effectiveness of the proposed framework, demonstrating substantially stronger performance on FSM5 under identical evaluation settings while maintaining lower computational cost. While Wav2Vec2 relies on large-scale pretraining and fine-tuning, the proposed CNN-ensemble framework offers a computationally efficient alternative that is well-suited for moderate-scale forest acoustic monitoring scenarios. Nevertheless, FSM5 remains a curated benchmark assembled from heterogeneous public datasets, and additional large-scale in-the-wild forest recordings will be necessary to further evaluate generalization under diverse environmental and recording conditions. As future work, the FSM5 dataset will be further expanded with additional realistic forest acoustic events to better capture the diversity and complexity of real-world soundscapes.

## Data Availability

The proposed FSM5 dataset is available at the following link: https://drive.google.com/drive/folders/1_vmRYJj7OqJDAIwkReMVapYdlAABt9Qt
